# Application of Response Surface Methodology for the Optimization of Basic Red 46 Dye Degradation in an Electrocoagulation–Ozonation Hybrid System

**DOI:** 10.3390/molecules30122627

**Published:** 2025-06-17

**Authors:** Nguyen Trong Nghia, Vinh Dinh Nguyen

**Affiliations:** 1 Faculty of Chemical and Environmental Technology, Hung Yen University of Technology and Education, Khoai Chau District, Hung Yen 17817, Vietnam; 2 Faculty of Natural Science and Technology, TNU-University of Sciences, Tan Thinh Ward, Thai Nguyen City 25000, Vietnam; vinhnd@tnus.edu.vn

**Keywords:** electrocoagulation–ozonation, Basic Red 46, azo dye removal, response surface methodology, pilot-scale study

## Abstract

The release of synthetic dyes like Basic Red 46 (BR46) from industrial wastewater has raised growing concerns due to their toxicity, long-term persistence, and resistance to standard biological treatment methods. In this work, we developed and tested a pilot-scale electrocoagulation–ozonation (EC–O) hybrid system aimed at removing BR46 from aqueous solutions. The system integrates electrocoagulation, using iron electrodes, with ozone-based advanced oxidation processes, facilitating a combination of coagulation, adsorption, and oxidative breakdown of dye molecules. The response surface methodology (RSM) with a central composite design (CCD) was applied to optimize the treatment process, focusing on five variables: current density, flow rate, ozone dosage, ozonation time, and initial dye concentration. The quadratic model exhibited strong predictive power, with an adjusted R^2^ of 0.9897 and a predicted R^2^ of 0.9812. The optimal conditions identified included a current density of 70 A/m^2^, flow rate of 1.6 L/min, ozone dose of 2.0 g/h, and an ozonation time of 20 min, achieving a predicted removal efficiency of 91.67% for a solution with BR46 at an initial concentration of 300 mg/L. Experiments conducted under these conditions confirmed the model’s reliability, with observed removal rates exceeding 90% and deviations under 2%. The EC–O system had a treatment capability of 26.19 L/h and an energy consumption of 3.04 kWh/m^3^. These findings suggest that the EC–O system is an effective and scalable option for treating dye-contaminated wastewater, offering faster and more efficient results than conventional techniques.

## 1. Introduction

The rapid growth of industrial sectors such as textiles, plastics, leather, printing, and cosmetics has led to a sharp increase in the global demand for synthetic dyes, particularly organic dyes, due to their vibrant colors, structural versatility, and chemical stability [1,2]. However, their widespread use has raised serious environmental concerns, especially when dye-laden effluents are discharged into aquatic systems. It is estimated that up to 15% of dyes used in industrial processes are lost to wastewater, producing highly colored, toxic, and non-biodegradable effluents [3]. Azo dyes, which account for more than 60% of all synthetic dyes, are particularly problematic due to their stable aromatic rings and azo (–N=N–) linkages, rendering them highly resistant to microbial degradation [4]. Even at low concentrations (a few mg/L), these dyes can severely impair light transmission, disrupt photosynthesis, and destabilize aquatic ecosystems [5]. Additionally, the degradation of azo dyes often yields aromatic amines—compounds known for their mutagenic, carcinogenic, and endocrine-disrupting properties—posing significant risks to both environmental and human health [6,7].

Basic Red 46 (BR46), a widely used cationic azo dye in the textile and paper industries, exemplifies these challenges. Its high solubility, intense color, and resistance to biodegradation make it a persistent pollutant in wastewater. Once released into water bodies, BR46 alters optical properties, inhibits primary reproduction, and contributes to ecological imbalance [8,9]. Moreover, its degradation intermediates are associated with notable toxicity and potential carcinogenicity. Thus, the development of efficient and robust treatment strategies for BR46 removal is not only an environmental priority but also critical for regulatory compliance and public health protection [10].

Ozonation is a powerful and environmentally friendly treatment method due to its high oxidation potential (2.07 V), operational simplicity, and scalability [11,12,13]. It has been widely applied for degrading persistent organic pollutants such as dyes and pharmaceuticals [14,15,16,17]. However, ozonation alone may produce toxic or non-biodegradable by-products, limiting its standalone effectiveness [18,19,20]. To overcome these drawbacks, hybrid systems combining ozonation with electrocoagulation (EC) have been proposed. The EC–O system synergistically integrates oxidative and adsorptive mechanisms. Iron electrodes release Fe^2+^ during EC, which reacts with ozone to generate hydroxyl radicals (2.8 V), enhancing oxidation. Concurrently, iron hydroxides form flocs that adsorb organic pollutants and their by-products, promoting effective pollutant removal through sedimentation [16,21,22]. This dual mechanism improves treatment efficiency, making EC–O suitable for complex wastewater matrices [23]. Several studies have demonstrated its efficacy across industrial effluents. For instance, EC–O achieved complete decolorization and COD reduction in distillery and industrial wastewater [16,19,24], while Behin et al. [25] reported 100% removal of Acid Brown 214. Similarly, color removal of up to 94% was achieved for C.I. Reactive Black 5 [26], outperforming ozonation or EC alone.

Response surface methodology (RSM) has become a valuable tool in real-world chemical and environmental engineering applications due to its ability to optimize complex processes efficiently and accurately [2]. In industrial and pilot-scale systems, where experimentation can be time-consuming, costly, or resource-intensive, RSM enables the identification of optimal operating conditions with minimal experimental runs. By constructing predictive mathematical models, RSM facilitates a deeper understanding of the relationships between input variables and system performance, allowing engineers to tune parameters for maximum efficiency. Importantly, RSM not only highlights the individual effects of process variables but also uncovers their interactive influences—factors often overlooked in conventional trial-and-error approaches [27,28]. This makes RSM particularly useful for scaling up laboratory findings to practical applications, ensuring process reliability, consistency, and economic feasibility. Its widespread adoption in wastewater treatment, pollutant removal, and chemical synthesis underscores its essential role in bridging the gap between theoretical design and operational implementation.

Pilot-scale studies play a critical role in bridging the gap between laboratory-scale research and full-scale industrial applications. While laboratory experiments provide valuable insights into fundamental mechanisms and treatment efficiencies, they often fail to capture the operational complexities encountered in real-world conditions. Pilot-scale investigations allow for the evaluation of process performance under more realistic scenarios, including variable influent characteristics, flow dynamics, and energy consumption patterns. These studies also help in assessing the durability, scalability, and cost-effectiveness of the treatment system, providing essential data for engineering design and process optimization [29]. Moreover, pilot trials facilitate the identification of potential operational challenges—such as fouling, sludge generation, and maintenance needs—that may not be evident at the bench scale. In the context of advanced hybrid processes like electrocoagulation–ozonation, pilot-scale evaluations are indispensable for validating treatment efficiency, optimizing process parameters, and ensuring the feasibility of large-scale implementation in wastewater treatment facilities.

In this study, a pilot-scale hybrid electrocoagulation–ozonation (EC–O) system was developed and evaluated for the enhanced removal of the cationic azo dye BR46 from synthetic wastewater. The performance of the system was assessed in terms of color removal and energy consumption under various operational conditions. Response surface methodology, based on a central composite design, was employed to optimize key process variables, including current density, rate flow, ozone dose, ozonation time, and initial BR46 concentration. The interactive effects of these variables on treatment efficiency were analyzed, and a predictive model was established to identify optimal operating conditions. The pilot-scale configuration of the EC–O system offers practical insights into its scalability and feasibility for real-world wastewater treatment applications.

## 2. Results and Discussion

### 2.1. Regression Model Evaluation

To identify the most suitable regression model for predicting BR46 removal using RSM, four models were evaluated: linear, two-factor interaction (2FI), quadratic, and cubic. The performance metrics of each model are summarized in Table 1. Among them, the quadratic model demonstrated superior predictive capability, exhibiting the highest adjusted R^2^ (0.9897) and predicted R^2^ (0.9812), indicating excellent accuracy and predictive reliability. Additionally, the model showed a non-significant lack-of-fit *p*-value (0.6174), suggesting a strong agreement between the predicted and experimental values with minimal unexplained variation.

Although the cubic model achieved a comparable adjusted R^2^ value (0.9889), it was found to be aliased, meaning that some higher-order terms were confounded and could not be interpreted independently, thereby limiting its practical usefulness. In comparison, while the linear and 2FI models were statistically significant based on their sequential model *p*-values (<0.0001 and 0.0083, respectively), they exhibited lower R^2^ values and a poorer overall fit. Based on these considerations, the quadratic model was selected as the most robust and statistically appropriate model for subsequent analysis and optimization.

The recommended model is described by the following second-order polynomial equation:

(1) $$Y=\beta_{0}+\sum_{i=1}^{k}\beta_{i}X_{i}+\sum_{i=1}^{k}\beta_{ii}X_{i}^{2}+\sum_{i<j}^{k}\beta_{ij}X_{i}X_{j}+\varepsilon$$

Here, Y denotes the predicted response (BR46 removal efficiency), β_0_ is the intercept term, β_i_ represents the coefficients of the linear terms, β_ii_ corresponds to the quadratic coefficients, β_ij_ indicates the interaction effects between variables X_i_ and X_j_, and ε is the random error term.

### 2.2. Model Adequacy and Significance Assessment

Analysis of variance (ANOVA) was performed to assess the adequacy and statistical significance of the quadratic model developed for predicting BR46 removal efficiency. As summarized in Table 2, the model was highly significant, with an F-value of 237.20 and a *p*-value less than 0.0001, indicating that the regression effectively accounts for the variability in the response. Among the main effects, current density (X_1_), ozone dose (X_3_), ozonation time (X_4_), and initial dye concentration (X_5_) exhibited strong and statistically significant influences (*p* < 0.0001). In contrast, flow rate (X_2_) did not significantly affect the response (*p* = 0.1079). Several two-way interaction terms—specifically X_1_X_2_, X_1_X_5_, X_2_X_4_, and X_2_X_5_—were also statistically significant (*p* < 0.05), suggesting that interactions between these variable pairs play a notable role in influencing BR46 removal efficiency. Additionally, the quadratic terms for current density (X_1_^2^), ozonation time (X_4_^2^), and dye concentration (X_5_^2^) were found to be significant (*p* < 0.05), indicating curvature in the response surface and the necessity of a second-order model. The non-significant lack-of-fit value (*p* = 0.6174) confirmed that the model adequately fit the experimental data, with no substantial unexplained variation. Furthermore, the low mean square error (MSE = 0.465) and high model significance affirm that the selected variables and model structure are appropriate for optimizing the BR46 removal process using the EC–O system.

The equation presenting the relationship between the response (BRE) and variables (parameters) can be written as follows:

(2) $$Y=\;78.02+\;7.75X_{1}+0.24X_{2}+4.83X_{3}+1.08X_{4}-2.66X_{5}+0.5937X_{1}X_{2}+0.2062X_{1}X_{3}\;+0.2119X_{1}X_{4}-0.4688X_{1}X_{5}+0.0756X_{2}X_{3}-0.5687X_{2}X_{4}-0.3994\;X_{2}X_{5}-\;0.1450X_{3}X_{4}-\;0.0481X_{3}X_{5}+0.3050X_{4}X_{5}-0.3244X_{1}^{2}-0.1356X_{2}^{2}-0.2106X_{3}^{2}-0.6619X_{4}^{2}+0.3269X_{5}^{2}$$

### 2.3. Model Diagnostics and Validation

**Figure 1:**
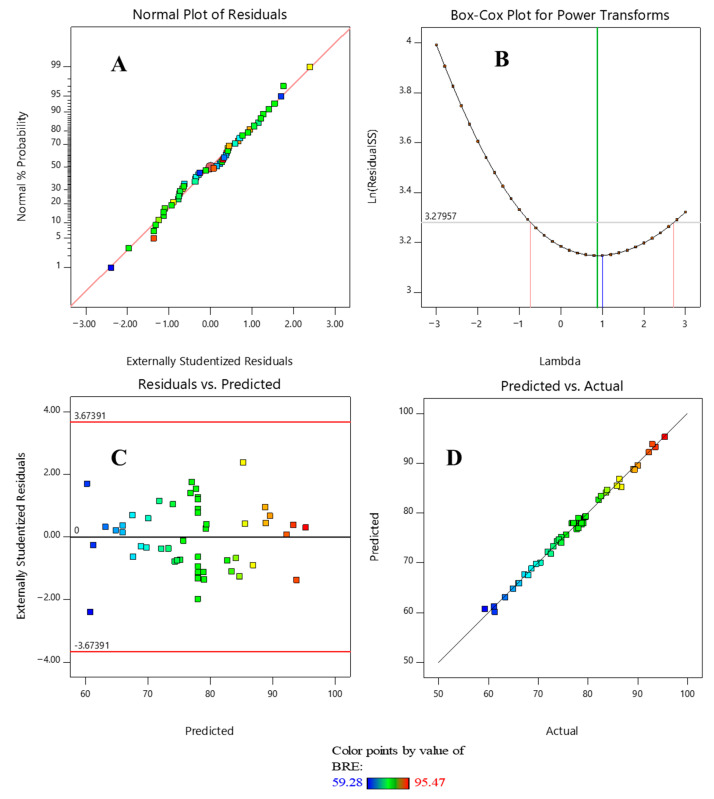
Normal plot of residuals (**A**), Box-Cox plot for power transform (**B**), residual versus predicted plot (**C**), and plot of predicted versus actual values of BR6 removal efficiency (**D**).

To validate the assumptions of the quadratic regression model, several diagnostic plots were analyzed, as shown in Figure 1. The normal probability plot of residuals (Figure 1A) was used to assess the normality of the error distribution. The residuals align closely along the reference line, indicating that the errors are approximately normally distributed. This supports the reliability of the ANOVA results and confirms that the model satisfies one of the fundamental assumptions of response surface methodology. The absence of pronounced curvature or deviation suggests that no data transformation is necessary, and the residuals do not display any systematic patterns that would compromise model validity. The Box–Cox plot (Figure 1B) was used to evaluate whether transformation of the response variable was required to meet the assumptions of normality and constant variance. The optimal lambda (λ) value, indicated by the green vertical line, is close to 1, and the 95% confidence interval (bounded by red lines) includes λ = 1. This confirms that the original, untransformed response variable is suitable for the model and that no transformation is needed. The plot of residuals versus predicted values (Figure 1C) was examined to test the assumptions of homoscedasticity and randomness. The residuals were distributed randomly around the zero line, with no discernible patterns or trends, and all values fell within the acceptable statistical bounds (±3.67). Although slightly larger residuals were observed at higher predicted values—suggesting mild heteroscedasticity—the variation was within acceptable limits, indicating that the model’s predictive capability remains robust. Finally, the correlation plot of predicted versus experimental values (Figure 1D) demonstrated a strong agreement between the model outputs and observed results. The data points clustered closely around the y = x line, with no significant outliers or systematic deviations, confirming the high predictive accuracy and reliability of the fitted quadratic model for describing and optimizing the BR46 removal process.

### 2.4. Interpretation of Three-Dimensional Response Surfaces

#### 2.4.1. Effect of Current Density on BR46 Removal Efficiency

**Figure 2:**
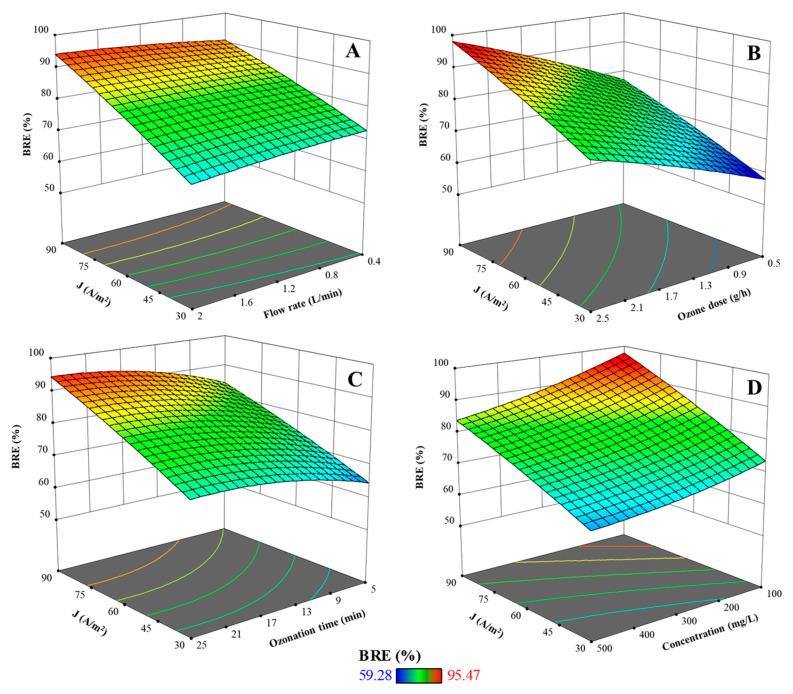
Three-dimensional response surface plots illustrating the effects of operational parameters on BR46 removal efficiency: (**A**) interaction between current density and flow rate; (**B**) interaction between current density and ozone dose; (**C**) interaction between current density and ozonation time; (**D**) interaction between current density and initial BR46 concentration.

As illustrated in Figure 2, current density emerged as the most influential parameter in the electrochemical ozonation process for BR46 removal. An increase in current density consistently enhanced BR46 removal efficiency across all experimental conditions. This improvement is attributed to the increased release of Fe^2+^ ions according to Faraday’s law [30,31]. These ions not only contribute to coagulation via floc formation but also catalyze the generation of hydroxyl radicals—potent oxidizing species that significantly accelerate dye degradation. ANOVA results confirmed the statistical significance of current density (*p* < 0.0001), and response surface plots consistently demonstrated its positive effect. The interaction between current density and flow rate (Figure 2A) was also statistically significant, though less dominant. Moderate flow rates slightly enhanced BRE by improving mass transfer, but excessive flow reduced residence time, thus limiting oxidation. The interaction between current density and ozone dose (Figure 2B), while not statistically significant in the ANOVA, revealed a near-linear additive effect, suggesting that both anodic and ozone-induced oxidation work synergistically. Similarly, the interaction with ozonation time (Figure 2C) showed enhanced removal with longer exposure, although diminishing returns were observed beyond a certain treatment duration. Finally, the interaction with dye concentration (Figure 2D) indicated that high current densities could partially offset the negative effects of higher dye loads, but only up to a point—extremely high concentrations still resulted in reduced efficiency. Overall, current density plays a central role in the process and must be carefully balanced for maximum system performance.

#### 2.4.2. Effect of Flow Rate on BR46 Removal Efficiency

**Figure 3:**
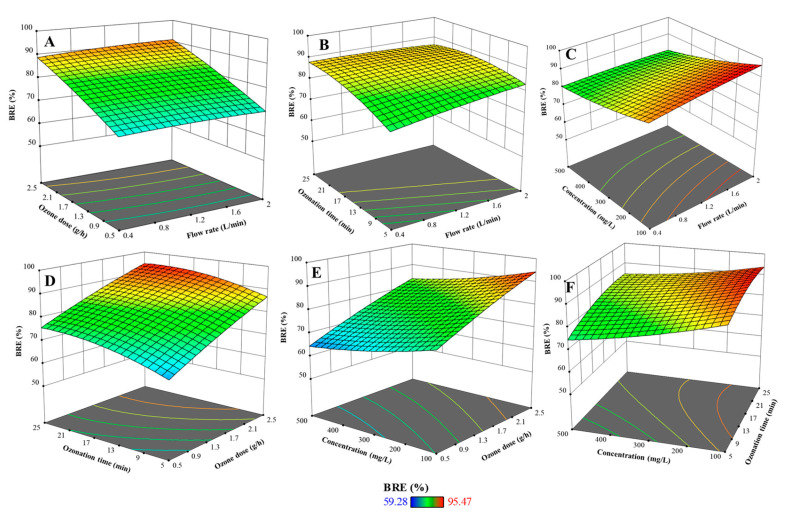
Three-dimensional response surface plots showing the interactive effects of operational variables on BR46 removal efficiency: (**A**) flow rate and ozone dose; (**B**) flow rate and ozonation time; (**C**) flow rate and initial BR46 concentration; (**D**) ozone dose and ozonation; (**E**) ozone dose and initial BR46 concentration; (**F**) ozonation time and initial BR46 concentration.

As presented in Figure 3A–C, the flow rate had a slight influence on BRE in the EC–O system. As the flow rate increased from low to moderate levels, a slight improvement in BRE was observed due to enhanced turbulence and mass transfer, promoting better interaction between reactive species and dye molecules [32,33]. However, excessively high flow rates shortened residence time, limiting oxidation efficiency. While ANOVA indicated that flow rate was not a statistically significant main factor (*p* = 0.1079), its interaction with other variables, particularly current density, was significant and impactful. The plots showed that flow rate affected outcomes related to ozone dose, ozonation time, and dye concentration. When paired with increasing ozone dose, higher flow rates slightly suppressed BRE due to reduced oxidant–pollutant contact time. Similarly, while extended ozonation times improved BRE, higher flow still hindered overall performance by limiting retention in the reactor. In combination with dye concentration, elevated flow rates exacerbated the negative effect of high pollutant loads. These findings suggest that although flow rate is not a primary driver of removal efficiency, it plays an important supportive role and must be optimized to balance mass transfer and residence time.

#### 2.4.3. Effect of Ozone Dose on BR46 Removal Efficiency

As presented in Figure 2B and Figure 3D,E, ozone dose was found to be a critical parameter in the EC–O system, with a significant and consistent impact on BR46 removal (*p* < 0.0001). As the ozone dose increased, a substantial improvement in BRE was observed across all conditions. This can be attributed to the increased availability of ozone and its secondary oxidants, such as hydroxyl radicals, which effectively degrade complex dye molecules [13,34]. Interaction analysis revealed that ozone dose works synergistically with other variables. In particular, the combination of high ozone dose and longer ozonation time significantly enhanced BRE, as extended contact time allowed for more complete oxidation. The interaction with dye concentration also demonstrated that increasing the ozone dose can partially compensate for higher pollutant loads. However, a saturation point was observed where additional ozone yielded diminishing returns, especially at very high concentrations. These results confirm that ozone dose must be aligned with the pollutant load to ensure effective and energy-efficient treatment.

#### 2.4.4. Effect of Ozonation Time on BR46 Removal Efficiency

Ozonation time plays a significant role in enhancing the removal efficiency of BR46 in the EC–O process. According to Figure 2B and Figure 3B,D,E, as the ozonation time increases, BR46 removal efficiency improves markedly, indicating that prolonged contact allows for more thorough oxidation of the dye molecules. Extended exposure facilitates the continuous generation of reactive oxidative species, including hydroxyl radicals and molecular ozone, which are essential for breaking down the complex structure of azo dyes like BR46 [13,14]. This trend is reflected in the response surface plots, where a steady rise in BRE is observed with longer ozonation durations. The ANOVA results further confirm the statistical significance of this parameter (*p* < 0.0001). However, beyond a certain point, the increase in efficiency begins to plateau, suggesting diminishing returns—likely due to the near-complete degradation of available dye or depletion of reactive species. These findings underscore the importance of optimizing ozonation time to ensure high treatment performance while minimizing unnecessary energy consumption and operational costs.

#### 2.4.5. Effect of Initial BR46 Concentration on BR46 Removal Efficiency

Initial BR46 concentration (Figure 3C,E,F) had a strong inverse effect on BRE, as confirmed by ANOVA (*p* < 0.0001). Higher concentrations increased the pollutant load, leading to a decline in removal efficiency due to the limited availability of oxidizing species. The response surface revealed a steady decrease in BRE with increasing dye concentration. Interactions with other variables, particularly current density and ozonation time, showed that increasing these parameters could partially offset the negative impact of high dye loads. Nevertheless, their compensatory effects diminished beyond a certain concentration threshold, where oxidant demand exceeded system capacity. These findings underscore the need to balance initial dye concentration with sufficient oxidant generation and residence time to maintain effective process performance.

### 2.5. Numerical Optimization of Process Parameters

In RSM, the optimization phase is critical for determining the most favorable operating conditions by systematically evaluating the influence of independent variables on the desired response. In this study, the primary objective was to maximize BR46 removal efficiency, which was assigned the highest importance level (5) within the desirability function framework. During the optimization process, the initial dye concentration was fixed at 200 mg/L, 300 mg/L, and 400 mg/L to simulate realistic treatment conditions, while other key variables—current density, flow rate, ozone dose, and ozonation time—were allowed to vary within their respective experimental ranges. The specific constraint settings and assigned importance levels for each parameter are detailed in Table 3.

The results of the numerical optimization are presented in Table 4. The selected optimal conditions are a current density of 70 A/m^2^, flow rate of 1.6 L/min, ozone dose of 2.0 g/h, and ozonation time of approximately 19–20 min at BR46 concentrations of 200, 300, and 400 mg/L. The predicted BRE values were 95.31%, 91.67%, and 88.93%, respectively. To evaluate the predictive performance of the RSM-derived quadratic model, experimental validation was conducted at three different BR46 concentrations (200, 300, and 400 mg/L) under optimized operating conditions. As summarized in Table 4, the predicted BRE values closely matched the corresponding experimental results, with deviations below 1.5% in all cases. At the lowest concentration (200 mg/L), the system achieved a maximum BRE of 96.15%, slightly exceeding the predicted value (95.31%). As expected, the removal efficiency gradually declined with increasing dye concentration, yet remained high at 91.54% and 87.92% for 300 and 400 mg/L, respectively. This slight decrease is attributed to the increased organic load requiring a greater amount of oxidizing species for complete degradation. The close agreement between predicted and experimental BRE values confirms the reliability and robustness of the model and the effectiveness of the optimized electro–ozone system across a range of BR46 concentrations.

To further confirm the degradation of BR46 under optimal treatment conditions, absorbance spectra of the sample with a BR46 concentration of 300 mg/L were recorded at different ozonation times (0, 10, and 20 min), as shown in Figure S1 (Supporting Information). Initially, a strong absorption peak centered at approximately 530 nm was observed, corresponding to the chromophoric azo group in the BR46 molecule. After 10 min of ozonation, the peak intensity significantly decreased, indicating the partial breakdown of the azo structure. At 20 min, the peak almost completely disappeared, suggesting extensive degradation of the dye. This result corroborates the high BR46 removal efficiency obtained from the model and highlights the effectiveness of the electro–ozonation process in disrupting the dye’s chromophore structure.

Table 5 presents a comparison of BR46 removal efficiencies (BRE) achieved by different treatment methods. Adsorption and photocatalytic processes both demonstrated high removal rates of approximately 90% but under notably different conditions. Adsorption required a prolonged contact time of 120 min at a concentration of 100 mg/L, while the photocatalytic process achieved similar efficiency within 20 min but only at a much lower dye concentration (40 mg/L). Biodegradation also showed promising results, with >90% removal, yet it required an extended treatment time of up to 120 h, limiting its practicality for rapid or large-scale applications. In contrast, the EC–O process developed in this study achieved a comparable or superior BRE of 91% at a significantly higher dye concentration (300 mg/L) within only 27.5 min. This highlights the advantages of the EC–O system in terms of treatment speed, scalability, and its robustness under high pollutant loads. These results affirm the efficiency and potential applicability of the EC–O system for advanced dye wastewater treatment.

### 2.6. Economic Evaluation and Scale-Up Considerations of EC–O System

The treatment capacity and energy efficiency of the EC–O system are critical factors in evaluating its feasibility for real-world wastewater applications. In the current pilot-scale configuration, the system was operated with a continuous flow rate of 1.6 L/min through the EC reactor and an ozonation time of 20 min, treating a total volume of 12 L per batch under optimized conditions. This corresponds to a throughput of approximately 26.19 L/h, indicating its suitability for semi-continuous or small-scale industrial applications.

Power consumption was calculated based on the applied current, voltage, and reaction time, taking into account both the electrocoagulation and ozonation units. The total energy consumption (E, kWh/m^3^) was estimated using the following equation:

(3) $$E=\frac{P_{EC}+P_{O}+P_{P}}{V}$$

where P_EC_, P_O_, and P_p_ (kW) are the energy consumption of the EC unit, ozonation unit, and pump, respectively, and V is the treated volume (m^3^). Under the optimized conditions—a current density of 70 A/m^2^, reaction time of 20 min, and ozone dose of 2.0 g/h—the specific energy consumption was found to be approximately 3.04 kWh/m^3^. Based on the average industrial electricity cost in Vietnam (~USD 0.084/kWh), the corresponding operational cost is estimated at ~USD 0.36/m^3^. This value is within a reasonable range when compared to other advanced oxidation processes, reflecting the system’s balance between high removal efficiency and manageable energy demand. The ozonation unit contributed significantly to the energy input, due to the continuous operation of the ozone generator. However, its role in enhancing pollutant degradation and reducing treatment time justified the additional energy cost. The hybrid system’s ability to achieve over 90% removal efficiency within 27.5 min further emphasizes its rapid treatment capacity, making it a competitive alternative to slower, more energy-intensive conventional methods [20,38].

### 2.7. Proposed Mechanism of BR46 Removal by Electrocoagulation–Ozonation Hybrid System

The removal of BR46 in the EC–O system is driven by the synergistic action of electrochemical coagulation and ozone-induced advanced oxidation processes. The proposed mechanism involves a combination of direct oxidation, generation of reactive oxygen species, and physical adsorption facilitated by iron hydroxide flocs.

In the electrocoagulation unit, the anodic dissolution of iron electrodes generates Fe^2+^ ions. These Fe^2+^ ions undergo hydrolysis in aqueous media to form ferrous hydroxide [Fe(OH)_2_], which further oxidizes to ferric hydroxide [Fe(OH)_3_] [39]. These hydroxide species act as coagulants, destabilizing dye molecules and promoting their aggregation into flocs that can be removed via sedimentation. Simultaneously, Fe^2+^ reacts with ozone, introduced in the second reactor, leading to the generation of hydroxyl radicals, a highly potent oxidizing species with a redox potential of 2.8 V [40]. Hydroxyl radicals non-selectively attack the chromophoric and auxochromic groups of the BR46 dye, breaking azo bonds (–N=N–), aromatic rings, and other functional groups. This leads to the formation of smaller, less toxic intermediates and ultimately to mineralization products such as CO_2_, H_2_O, and inorganic ions.

In addition to oxidation, coagulation and adsorption contribute significantly to dye removal. The in situ-formed iron hydroxides possess a high surface area, allowing for the adsorption of dye molecules and their partially oxidized intermediates. These flocs settle out of the solution, facilitating the physical removal of pollutants alongside chemical degradation. The synergy between electrocoagulation and ozonation processes enhances treatment efficiency by maintaining a continuous supply of oxidants and coagulants. Electrocoagulation supports ozone decomposition and radical formation, while ozonation enhances the oxidative breakdown of organic structures that are not effectively removed by coagulation alone. The EC–O system integrates multiple removal pathways—oxidation, coagulation, and adsorption—resulting in efficient and rapid degradation of BR46. The combined mechanism ensures high removal efficiency, reduced sludge production, and minimal formation of harmful by-products, making it a viable solution for treating dye-laden wastewater.

## 3. Materials and Methods

### 3.1. Chemicals

All chemicals used in this study, including sodium chloride (NaCl), hydrochloric acid (HCl), and sodium hydroxide (NaOH), were of analytical grade and obtained from Merck (Rahway, NJ, USA). These reagents were used as received, without any further purification. The cationic dye Basic Red 46 (BR46, C_18_H_21_BrN_6_) was purchased from Macklin (Shanghai, China).

### 3.2. Ozone–Electrocoagulation System

**Figure 4:**
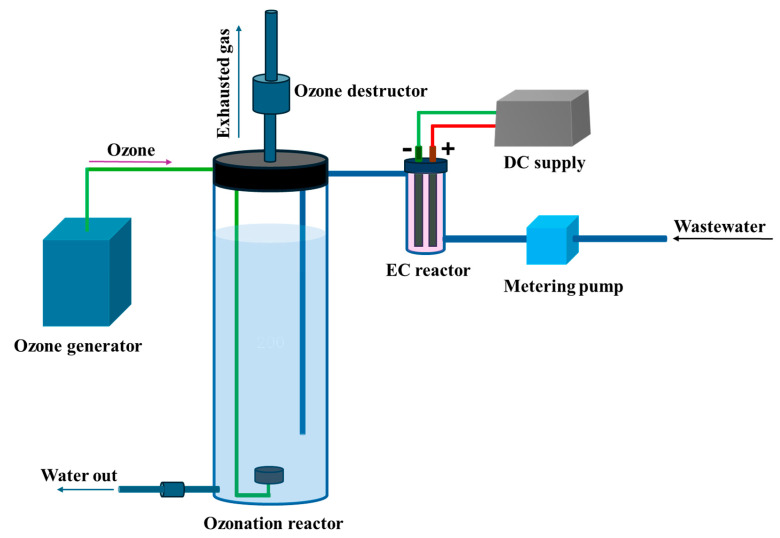
Schematic of pilot-scale EC–O system used for BR46 removal.

The EC–O system used for the removal of BR46 is schematically illustrated in Figure 4. The setup consists of two main units: an EC reactor and an ozonation reactor. The EC reactor, with a working volume of 0.3 L, was connected to a metering pump through a PVC pipeline. Iron plates (99.5% Fe), 21 cm in length, 4.5 cm in width, and 0.5 cm in thickness, were employed as electrodes. The working area of each electrode was 200 cm^2^. These electrodes were connected to a DC power supply capable of delivering a current of 0–10 A and a voltage range of 0–40 V. The effluent from the EC reactor was transferred to the ozonation reactor, which had a capacity of 15 L and was also connected via PVC piping. Ozone was generated using a commercial ozone generator (Model RO-10GH, HSVN, Hanoi, Vietnam) and continuously introduced into the reactor through ozone diffusers to ensure uniform gas distribution and effective contact with the wastewater.

In each experiment, 12 L of the BR46 dye solution was adjusted to a pH in the range of 6–7 and a conductivity of about 14.5 mS/cm. Then, this solution was pumped into the EC reactor. The solution flowed through the EC unit at a controlled flow rate and was subsequently transferred to the ozonation reactor. During this pumping phase, a specified current density was applied to the EC reactor. Ozone was then introduced into the ozonation reactor at a predetermined dose for a defined reaction time. At a specific interval, a 5 mL aliquot of the treated solution was immediately collected and mixed with sodium thiosulfate (Na_2_S_2_O_3_) to quench any residual ozone. The sample was then filtered, and the residual concentration of BR46 was measured using a UV–Vis spectrophotometer (V-770, Jasco, Tokyo, Japan) at a wavelength of 530 nm. To ensure reproducibility and accuracy, all experiments were performed in triplicate.

The removal efficiency of Basic Red 46 (BRE, %) was calculated using the following equation:

(4) $$BRE=\frac{C_{o}-C}{C_{o}}\times 100$$

where C_o_ (mg/L) and C (mg/L) are the BR46 concentration at the beginning and after treatment. Ozone dose was determined using the standard potassium iodide wet-chemistry method, which quantifies ozone concentration based on its oxidative reaction with iodide ions in solution [41].

### 3.3. Experimental Design

Before the experimental design, preliminary tests were conducted to identify key factors influencing the BR46 removal process and to establish appropriate value ranges for each. The preliminary screening results are provided in the Supporting Information (Tables S1–S3). Based on these results, five independent variables including current density, flow rate, ozone dose, ozonation time, and initial BR46 concentration were selected for optimization. The ranges of the five independent variables were selected based on preliminary tests and the previous literature. Table 6 summarizes the coded and actual values for each factor used in the CCD model. Each variable was evaluated at five coded levels: low (−1), center (0), high (+1), and at two axial points (−α and +α).

## 4. Conclusions

The pilot-scale hybrid EC–O system was successfully developed and optimized for the effective removal of the cationic azo dye BR46 from synthetic wastewater. The combined process leveraged the synergistic effects of electrocoagulation and ozonation, integrating oxidative degradation, coagulation, and adsorption mechanisms. RSM with a CDC was employed to systematically investigate the effects of key operational parameters, including current density, flow rate, ozone dose, ozonation time, and initial dye concentration. The quadratic regression model developed through RSM demonstrated excellent predictive accuracy. Numerical optimization identified the optimal operating conditions—a current density of 70 A/m^2^, flow rate of 1.6 L/min, ozone dose of 2.0 g/h, and ozonation time of 20 min—resulting in a predicted BRE of 91.67%. Experimental validation confirmed the model’s accuracy, with removal efficiencies consistently above 90%. The EC–O system had a treatment capability of 26.19 L/h and an energy consumption of 3.04 kWh/m^3^. The EC–O process achieved significantly higher degradation efficiency in a shorter time frame, making it a promising and sustainable technology for treating dye-contaminated wastewater. The findings indicate the potential of hybrid electrocoagulation–ozonation systems in environmental remediation and demonstrate the value of RSM as a powerful tool for process optimization. However, to apply to real wastewater, the toxicity evaluation of treated effluents, analysis of intermediate by-products, and assessment of ozone utilization efficiency need to be performed to ensure environmental safety. Moreover, evaluating the EC–O process under real textile wastewater conditions, where multiple dye types and matrix complexity may influence treatment efficiency, will allow for a more comprehensive assessment of agglomeration dynamics, sludge generation, and process capability in practical settings.

## Figures and Tables

**Table 1:** Evaluation of linear, 2FI, quadratic, and cubic models for predicting BR46 removal efficiency.

Source	Sequential *p*-Value	Lack of Fit *p*-Value	Adjusted R^2^	Predicted R^2^
Linear	<0.0001	0.1061	0.9748	0.9705
2FI	0.0083	0.2441	0.9827	0.9781
Quadratic	0.0009	0.6174	0.9897	0.9812
Cubic	0.6139	0.5137	0.9889	0.9547

**Table 2:** ANOVA for the quadratic model predicting BR46 removal efficiency.

Source	Sum of Squares	df	Mean Square	F-Value	*p*-Value
Model	3808.74	20	190.44	237.20	<0.0001
X_1_-J	2399.40	1	2399.40	2988.54	<0.0001
X_2_-Flow rate	2.21	1	2.21	2.75	0.1079
X_3_-Ozone dose	931.23	1	931.23	1159.87	<0.0001
X_4_-ozonation time	129.60	1	129.60	161.42	<0.0001
X_5_-BR46Concentration	283.02	1	283.02	352.52	<0.0001
X_1_X_2_	11.28	1	11.28	14.05	0.0008
X_1_X_3_	1.36	1	1.36	1.70	0.2031
X_1_X_4_	1.44	1	1.44	1.79	0.1914
X_1_X_5_	7.03	1	7.03	8.76	0.0061
X_2_X_3_	0.1830	1	0.1830	0.2279	0.6366
X_2_X_4_	10.35	1	10.35	12.89	0.0012
X_2_X_5_	5.10	1	5.10	6.36	0.0174
X_3_X_4_	0.6728	1	0.6728	0.8380	0.3675
X_3_X_5_	0.0741	1	0.0741	0.0923	0.7634
X_4_X_5_	2.98	1	2.98	3.71	0.0640
X_1_^2^	3.37	1	3.37	4.19	0.0497
X_2_^2^	0.5886	1	0.5886	0.7331	0.3989
X_3_^2^	1.42	1	1.42	1.77	0.1940
X_4_^2^	14.02	1	14.02	17.46	0.0002
X_5_^2^	3.42	1	3.42	4.26	0.0481
Residual	23.28	29	0.8029		
Lack of Fit	17.14	22	0.7791	0.8879	0.6174
Pure Error	6.14	7	0.8775		
Cor Total	3832.03	49			

**Table 3:** Criteria of parameters and BRE used for optimization using numerical optimization.

Parameters	Goal	Lower Limit	Upper Limit	Lower Weight	Upper Weight	Importance
J (A/m^2^)	is in range	30	70	1	1	3
Flow rate (L/min)	is in range	0.8	1.6	1	1	3
Ozone dose (g/h)	is in range	1	2	1	1	3
Ozonation time (min)	is in range	10	20	1	1	3
Concentration (mg/L)	200, 300, 400	200	400	1	1	3
BRE (%)	maximize	59.28	100	1	1	5

**Table 4:** Optimal conditions, predicted BRE, and experimental BRE under optimal conditions.

J (A/m^2^)	Flow Rate (L/min)	Ozone Dose (g/h)	Ozonation Time (min)	Concentration (mg/L)	Predicted BRE (%)	Experimental BRE (%)
70.00	1.600	2	18.760	200	95.31	96.15
70.00	1.600	2	20.000	300	91.67	91.54
69.97	1.110	2	20.000	400.	88.93	87.92

**Table 5:** Comparison of the BR46 removal efficiency of different methods.

Methods	BR46 Concentration	Time (min)	BRE (%)	Ref.
Adsorption	100 mg/L	120 min	~90%	[35]
Photocatalysis	40 mg/L	20 min	~90%	[36]
Biodegradation	100 mg/L	120 h	>90%	[37]
EC–O	300 mg/L	27.5 min	~91%	This study

**Table 6:** Independent variables and their levels selected for response surface modeling using CCD.

Independent Variables	Range				
	−α	−1	0	+1	+α
Current density (A/m^2^)	10	30	50	70	90
Flow rate (L/min)	0.4	0.8	1.2	1.6	2.0
Ozone dose (g/h)	0.5	1.0	1.5	2.0	2.5
Ozonation time (min)	5	10	15	20	25
BR46 concentration (mg/L)	100	200	300	400	500

## Data Availability

The original contributions presented in this study are included in the article/Supplementary Materials. Further inquiries can be directed to the corresponding author.

## Supplementary Materials

The following supporting information can be downloaded at https://www.mdpi.com/article/10.3390/molecules30122627/s1.
