# Supplementary material for: Application of Response Surface Methodology for the Optimization of Basic Red 46 Dye Degradation in an Electrocoagulation–Ozonation Hybrid System
Source: Molecules. 2025 Jun 17;30(12):2627. doi: 10.3390/molecules30122627 (PMC12195645; doi:10.3390/molecules30122627)
Supplement: Supplementary file 1 [file molecules-30-02627-s001.zip › molecules-3681798-supplementary.pdf]

Supporting Information

Application of Response Surface Methodology for the Optimization of Basic Red 46 Dye Degradation in an Electrocoagulation–Ozonation Hybrid System

Nguyen Trong Nghia [1,*] and Vinh Dinh Nguyen [2]

[1] Faculty of Chemical and Environmental Technology, Hung Yen University of Technology and Education, Khoai Chau District, Hung Yen 17817, Vietnam
[2] Faculty of Natural Science and Technology, TNU-University of Sciences, Tan Thinh Ward, Thai Nguyen City 25000, Vietnam; vinhnd@tnus.edu.vn
* Correspondence: nguyentrongnghia@utehy.edu.vn

To identify the most influential parameters affecting the removal efficiency of BR46, screening experiments were conducted using a two-level factorial design. Seven independent variables were initially considered: current density, pH, electrode distance, flow rate, ozone dose, ozonation time, and initial dye concentration. The values of each variable are presented in Table S1.

Table S1. Values of seven variables used for screening experiments

Variables	Units	Low (-1)	High (+1)
Current density	A/m^2	30	70
pH	-	4	9
Electrode distance	mm	10	20
Flow rate	L/min	0.8	1.6
Ozone dose	g/h	1	2
Ozonation time	min	10	20
Concentration	Mg/L	200	400

Table S2. A total of 16 experimental runs were performed and the values of BRE

Run	Current density (A/m^2)	pH	Distance (mm)	Flow rate (L/min)	O_3 dose (g/h)	O_3 time (min)	Conc. (mg/L)	BRE (%)
1	70	9	20	1.6	2.0	20	400	76.82
2	30	4	20	0.8	2.0	10	400	74.15
3	70	4	20	1.6	1.0	10	400	66.3
4	30	9	20	1.6	2.0	10	200	74.94
5	30	9	20	0.8	1.0	20	400	69.72
6	70	9	10	0.8	2.0	10	400	84.44
7	30	4	20	1.6	1.0	20	200	73.68
8	70	9	10	1.6	1.0	20	200	79.41
9	30	9	10	1.6	1.0	10	400	64.25
10	30	9	10	0.8	2.0	20	200	82.39
11	70	4	10	1.6	2.0	10	200	82.72
12	30	4	10	1.6	2.0	20	400	70.18
13	70	4	20	0.8	2.0	20	200	91.23
14	70	9	20	0.8	1.0	10	200	77.85
15	30	4	10	0.8	1.0	10	200	68.22
16	70	4	10	0.8	1.0	20	400	80.79

An analysis of variance (ANOVA) was conducted to evaluate the significance of each independent variable on the BR46 removal efficiency (BRE). The analysis of variance (ANOVA) results provided in the table reveal the relative significance of each experimental factor on the response variable (BRE – BR46 removal efficiency). Among the seven tested factors, current density (F = 25.73, $p = 0.0010$), ozone dose (F = 21.46, $p = 0.0017$), concentration of BR46 (F = 12.82, $p = 0.0072$), flow rate (F = 10.96, $p = 0.0107$), and ozonation time (F = 6.57, $p = 0.0335$) were found to have statistically significant effects ($p < 0.05$) on the dye removal efficiency. In contrast, pH and electrode distance exhibited no significant impact on the response ($p > 0.5$), indicating that they can be excluded from further optimization studies. These results validate the selection of the five most influential parameters (current density, flow rate, ozone dose, ozonation time, and concentration) for inclusion in the response surface methodology (RSM) optimization.

Table S3. An analysis of variance of the variables

Source	Sum of Squares	df	Mean Square	F-value	p-value
Current density (A/m^2)	240.48	1	240.48	25.73	0.0010
pH	0.4064	1	0.4064	0.0435	0.8400
Electrode distance (mm)	3.72	1	3.72	0.3975	0.5460
Flow rate (L/min)	102.47	1	102.47	10.96	0.0107
Ozone dose (g/h)	200.58	1	200.58	21.46	0.0017
Ozonation time (min)	61.43	1	61.43	6.57	0.0335
Concentration (mg/L)	119.85	1	119.85	12.82	0.0072

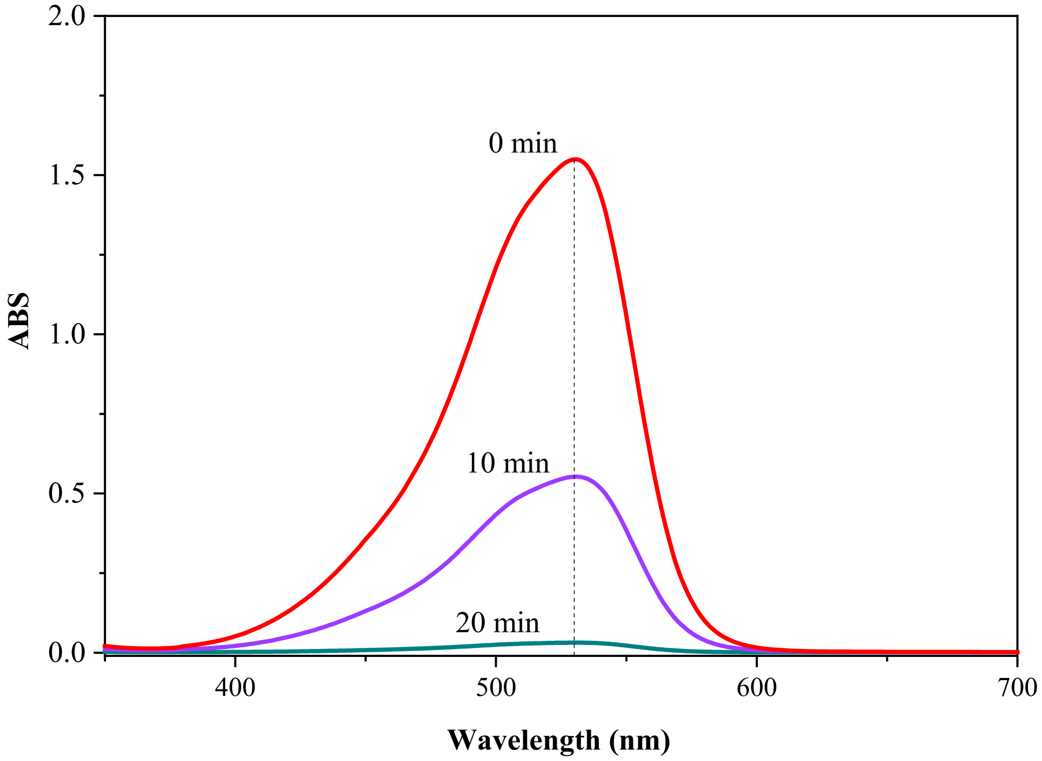

Figure S1. Absorbance spectra of the BR46 solution of 300 mg/L at the beginning (red line), after 10 minutes (purple line), and after 20 minutes (green line). The solutions were diluted 6 times before the analysis
